# Performance Indicators in Young Elite Beach Volleyball Players

**DOI:** 10.3389/fpsyg.2019.02712

**Published:** 2019-12-12

**Authors:** José Antonio Pérez-Turpin, Luis María Campos-Gutiérrez, Carlos Elvira-Aranda, María José Gomis-Gomis, Concepción Suárez-Llorca, Eliseo Andreu-Cabrera

**Affiliations:** Research Group on Physical Activity Sciences and Sport, University of Alicante, Alicante, Spain

**Keywords:** beach volleyball, technical, match analysis, gender, age group

## Abstract

**Purpose:**

The aim of this study was to analyze tactical and technical behavior across different ages and genders in young, elite beach volleyball players.

**Methods:**

Forty teams from the Under-18, Under-20, Under-21, and Under-22 from semifinals and finals of the 2016 World Championships and the 2016 European Championship were analyzed. The sample was composed of 69 sets. The variables studied included: Rally time, set time, match time, serve efficacy (standing serve, floating serve, and jump serve), setting efficacy (forearm, overhand, other, and 2nd attack), attack efficacy, and block efficacy. Student’s *t* and Mann–Whitney *U*-tests were used to analyze specific differences between categories.

**Results:**

Significant differences (*p* < 0.05) in the pass performance, forearm pass in men (from 78.2 to 49.1%), and overhand pass in men (from 12.2 to 40.45%). In addition, in women forearm pass (from 88.5 to 76.3%) and overhand pass in women (from 1.2 to 9.35%). No significant differences in the effectiveness of attack, rally time, serve, and block efficacy.

**Conclusion:**

Tactical considerations and gender-specific differences in technical variables may be important for training in young players.

## Introduction

The goal of sport training is to prepare athletes for the demands of games (García-de-Alcaraz et al., 2016). Coaches need reference values for these demands to guide and plan athletes’ training and development (Sheppard et al., 2009). Most of the information available about this topic is related to the physical demands on professional or international players (García-de-Alcaraz et al., 2016). Coaches have always been concerned with optimizing their teams’ performance given that this is an inherent part of competition (Hughes and Franks, 2004, 2008).

Game analysis in sport has become increasingly important for players over recent years (Koch and Tilp, 2009). There is, however, less information available on the demands of games for different age groups. This information is necessary to provide proper, specific training at the different stages of an athlete’s development, and to avoid centering training on physical aspects (Zemková et al., 2017).

Each sport has its own rules and characteristics which make it necessary to have specific reference values to guide athletes’ training in different stages of their development (García-de-Alcaraz et al., 2016). Beach volleyball, a team sport played by two teams of two players on a sand court (Natali et al., 2017). Beach volley game is divided in two phases: side out and counterattack. The sequence of actions in beach volleyball are: serve, serve reception, set, attack, block, and dig (Giatsis and Zahariadis, 2008). The side out phase includes: serve reception, setting, and attack. The counterattack includes: block, dig, set, and attack (Costa et al., 2012).

Beach volley became an Olympic sport in 1996 and has been growing exponentially ever since (Couvillon, 2004). For example, the 2006 World Tour featured 29 tournaments around the world and included 11–20 events on average. The characteristics of the game have been previously studied (Hömberg and Papageorgiou, 1994; Hansen, 2002; Papageorgiou and Hömberg, 2004), but sports are in constant evolution and it is necessary to analyze them on a permanent basis. In 2001, rules on court size and the scoring system changed, which led authors to research how this affected the game. Some studies investigated court size (Giatsis and Tzetzis, 2003; Giatsis et al., 2003; Kröger, 2006; Ronglan and Grydeland, 2006) and others focused on the scoring system (Giatsis et al., 2005; Kröger, 2006; Ronglan and Grydeland, 2006). The rule changes did not produce positive results in the view of the Fédération Internationale de Volleyball (FIVB). Furthermore, some studies analyzed technical and tactical aspects such as side out and counter-attack phases, receptions, and differences between winning and losing teams (Giatsis and Tzetzis, 2003; Michalopoulou et al., 2005; Giatsis et al., 2015).

There is also a need for studies comparing tactical and technical characteristics in female and male players as there might be potential differences influencing the game. Therefore, the aim of this study was to analyze tactical and technical behavior across different ages and genders in young, elite beach volleyball players.

## Materials and Methods

### Subjects

The participants of this study were 80 male and female volleyball players from 40 Under-18, Under-20, Under-21, and Under-22 teams. A total of 69 sets and 2,552 rallies (1,279 from women’s competitions and 1,273 from men’s competition) were analyzed. The sample included 69 sets of Under-18, Under-19, Under-20, Under-21, and Under-22 teams (536, 465, 512, 531, and 508 rallies for each category, respectively). The sets analyzed were from semi-finals and finals of the 2016 World Championships and the 2016 European Championship for Under-18, Under-19, Under-20, Under-21, and Under-22 teams. Due to the fact that a beach volleyball game is over when one of the teams wins two sets, a game can last two or three sets. The video recording of the matches was obtained from public web platforms. The location of the cameras was not fixed and depended on local factors. Therefore, the analysis of the videos was constrained by different levels of recording (Koch and Tilp, 2009; Koch et al., 2009; Natali et al., 2017; Giatsis et al., 2019). The recording process did not affect the behavior of players/teams as it is non-invasive and a common way to monitor competitions. The study was approved by the Bioethics Commission of the University of Alicante, and complied with the ethical principles stated by the Declaration of Helsinki.

### Design

An observational, descriptive, and correlational design was implemented together with a notational analysis in order to assess the different features of the technical–tactical elements analyzed. Variables were obtained through an observational methodology, which provides a quality method for analysis (Anguera, 2003). The aim of approaches such as observational methodology is to model sequences of actions to gain a deeper insight into the tactical behavior of teams (Koch and Tilp, 2009). Data were collected using an observational category system. The dependent variables studied included: (a) Rally time (average time between the start and end of the rally): play time, rest time, and ratio; (b) set time (average time between the start and end of the set): Time S1–S2 (average time between the start of set 1 and end of set 2), total time (average time between the start of set 1 and end of set 3), and number of rallies (average number of rallies in set); (c) match time (average time between the start and end of the match): play time (total average time between the start and end of the rally), passive time (total average time between the end and star of the rally), rest time (average time between sets, time outs, and technical times), total time (average time of match), and numbers of rally (average number of rallies in match); (d) serve efficacy: standing serve, floating serve, and jump serve; (e) setting efficacy: forearm, overhand, other, and 2nd attack; (f) attack efficacy; and (g) block efficacy. Three levels were established for efficacy: (+) win point, (−) lose point, and neutral, which permits a subsequent attack by the opposing team. Ball action performance was measured in relation to their effect on the rally and the opponent’s possibility to continue playing. Game phase performance was measured in relation to whether the team playing that phase won or lost the rally. The independent variables considered in our study were (a) gender: male or female; (b) competition team: Under-18, Under-19, Under-20, Under-21, and Under-22.

### Procedures

Matches were obtained from web platforms. Different levels of recording were found. We made sure that at least the official court area (16 × 8 m) was on camera in order to allow full viewing of actions. Rally time, set time, match time, serve efficacy (standing serve, floating serve, and jump serve), setting efficacy (forearm, overhand, other, and 2nd attack), attack efficacy, and block efficacy were collected by a single observer using Dartfish TeamPro 5.0 (Guo, 2018) and LongoMatch 1.0 free software (López-González and Miarka, 2013). The observer had a degree in sport science and over 2 years of experience in coaching and performance analysis in volleyball.

Before starting data collection, the observer completed specific training. Intra-observer reliability was calculated before and at the end of the process using two displays (Davis et al., 2008) using the following mathematical formula (Hughes, 2004):

Erm(%)=(Σ(mod[V1-V2])/Vmedia)*100

where V1 are the frequencies of the first visualization display and V2 the frequencies of the second visualization; media shows the average of the two frequencies of visualization, and mod is the module.

The reliability on the intra-observer analysis obtained a margin of error of <5%, i.e., within acceptable margins of error in display and analysis (James et al., 2007). Following Bakeman and Quera (2011), we carried out a Cohen’s kappa using SPPS Statistics 18 for inter-observer reliability, and we reached an inter-observer concordance value of 0.95, a virtually perfect value (Landis and Koch, 1977).

### Statistical Analysis

Descriptive (mean and standard deviation) and inferential tests were carried out using SPPS Statistics 18. We carried out the Kolmogorov–Smirnov test to analyze normality of data. The Mann–Whitney *U*-test with a Bonferroni *post hoc* (*p* < 0.01) was carried out to analyze variables with non-parametric distributions. Variables with parametric distributions were analyzed with Student’s *t*-distribution (*p* < 0.05).

## Results

Table 1 shows gendered timing characteristics across the different teams. In the case of women, work time and play time are constant in all teams. In the case of men, both decrease with age.

**TABLE 1:** Gendered timing characteristics.

	**U22 M^1^**	**U22 F^2^**	**U21 M**	**U21F**	**U20 M**	**U20F**	**U19 M**	**U19F**	**U18 M**	**U18F**
**Rally (s)**										
Work time	5.13	6.03	5.97	5.43	6.23	6.07	6.07	6.01	5.93	6.00
Rest time	17.74	17.50	18.16	16.63	17.13	15.41	15.41	14.08	15.55	17.50
Ratio	1:4.48	1:3.79	1:4.03	1:4.26	1:3.69	1:3.59	1:3.59	1:3.31	1:3.68	1:3.95
**Set (min)**										
Time S1–S2	16:49(1:19)	17:44(4:43)	18:44(1:15)	16:38(0:55)	18:45(2:41)	15:04(1:23)	17:13(2:50)	14:42(1:24)	19:04(5:02)	16:09(1:47)
Total time	16:49(1:19)	15:56(5:11)	18:20(1:32)	15:56(1:53)	18:45(2:41)	14:20(2:06)	16:22(3:26)	14:42(1:24)	18:32(4:48)	16:05(1:38)
Rallies	37.33 (3.01)	35.5 (8.19)	38.43 (4.08)	37.43 (4.54)	39.17 (4.92)	34.63 (4.63)	35.86(6.23)	34.14 (2.66)	42.0 (10.50)	34.57 (6.45)
**Match (min)**										
Play time	6:23(0:25)	9:31(1:47)	8:56(2:05)	7:54(3:12)	8:08(0:47)	8:56(3:11)	8:28(2:13)	7:09(0:22)	9:41(3:02)	8:04(1:09)
Passive time	22:04(1:53)	27:37(10:23)	27:09(6:29)	24:57(3:22)	22:22(4:23)	24:13(5:34)	21:30(6:19)	16:44(1:36)	25:24(5:27)	23:32(6:33)
Rest time	6:32(0:40)	8:30(2:25)	8:51(2:27)	8:43(2:45)	7:38(1:29)	7:34(2:16)	8:54(1:30)	6:57(0:46)	10:13(4:23)	8:23(2:00)
Total time	35:00(2:28)	45:38(14:32)	44:56(10:48)	41:34(9:19)	38:08(3:56)	40:44(10:14)	38:52(9:48)	30:50(2:34)	45:17(11:09)	39:59(9:30)
Rallies	74.66 (4.16)	94.66 (23.59)	89.66 (15.89)	87.33 (9.19)	78.33 (7.77)	92.33 (17.79)	83.66 (15.89)	71.33 (2.34)	98 (28.84)	80.66 (15.82)

*1. Male; 2. Female.*

**FIGURE 1:**
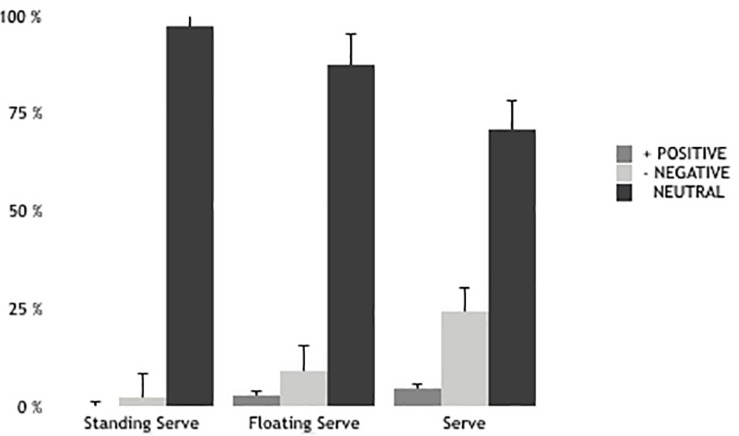
Effectiveness of serve in men.

**FIGURE 2:**
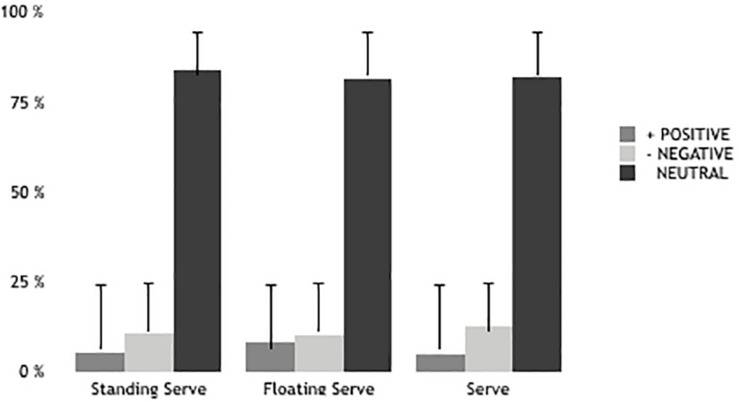
Effectiveness of serve in women.

The results showed significant gendered differences in effectiveness of serve (*z* = −3.540; *p* = 0.001) but no significant differences in errors (*z* = −0.762; *p* = 0.446) (Figures 1, 2). Therefore, in terms of serve we only found significant accuracy differences between U-18 and U-21 (*z* = −2.031; *p* = 0.042) and significant error differences between U-19 and U-22 (*z* = −2.986; *p* = 0.003), U-21 and U-22 (*z* = −2.315; *p* = 0.021).

As for setting (Table 2), we observed a significantly higher use of the overhand pass in men (*z* = −19.768; *p* = 0.001) in relation to forearm pass (*z* = −13.722; *p* = 0.001) and also a significantly higher use the overhand pass was observed at U-18 and U-19 (*z* = −7.253; *p* = 0.001), U-18 and U-21 (*z* = −9.147; *p* = 0.001), U-18 and U-22 (*z* = −7.615; *p* = 0.001), U-19 and U-20 (*z* = −6.695; *p* = 0.001), U-19 and U-21 (*z* = −1.993; *p* = 0.046), U-20 and U-21 (*z* = −8.580; *p* = 0.001), and U-20 and U-22 (*z* = −7.0556; *p* = 0.001). In terms of the forearm pass, differences were observed between U-18 and U-19 (*z* = −6.227; *p* = 0.001), U-20 (*z* = −2.304; *p* = 0.021), U-21 (*z* = −8.708; *p* = 0.001), U-22 (*z* = −6.866; *p* = 0.001), U-19 and U-20 (*z* = −3.940; *p* = 0.001), U-19 and U-21 (*z* = −2.491; *p* = 0.013), U-20 and U-21 (*z* = −6.432; *p* = 0.001), and U-20 and U-22 (*z* = −4.494; *p* = 0.001).

**TABLE 2:** Distribution types of setting across age and gender.

	**U22**	**U22**	**U21**	**U21**	**U20**	**U20**	**U19**	**U19**	**U18**	**U18**
**Setting**	**M^1^**	**F^2^**	**M**	**F**	**M**	**F**	**M**	**F**	**M**	**F**
**Technique (%)**										
Forearm	49.1	76.3	44.4	81.3	70.1	85.3	54.8	82.6	78.2^3^	88.5
Overhand	40.35^3^	9.3^3^	44.9^3^	0.8	15.1	0.0	35.6^3^	2.5	12.2	0.0
Other	0.9	1.1	1.8	1.5	1.3	2.5	2.2	2.5	1.4	3.0
2° attack	9.7	13.3	8.9	16.5	13.5	12.2	7.4	12.3	8.2	8.5

*1. Male; 2. Female; and 3. Significant differences.*

**FIGURE 3:**
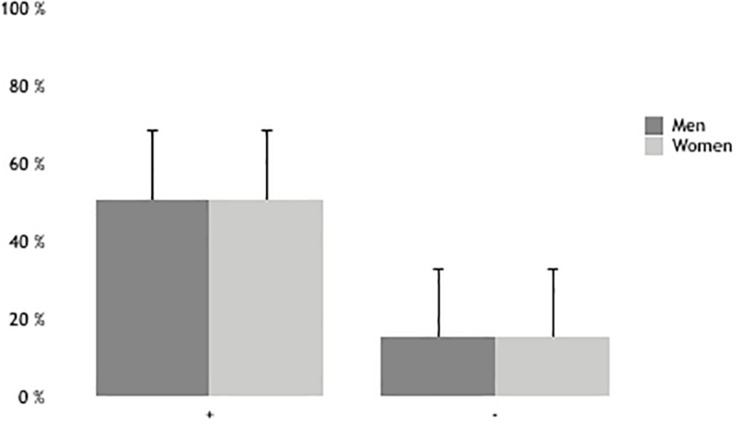
Gendered attack effectiveness.

Figure 3 shows gendered attack effectiveness, with no significant differences (*z* = −0.248; *p* = 0.804). In addition to this, we found accuracy differences in U-18 and U-20 (*z* = −2.146; *p* = 0.032), U-18 and U-21 (*z* = −2.464; *p* = 0.014), U-19 and U-20 (*z* = −2.378; *p* = 0.017), U-19 and U-21 (*z* = −2.307; *p* = 0.021), U-20 and U-21 (*z* = −4.725; *p* = 0.001), U-20 and U-22 (*z* = −2.785; *p* = 0.005). In terms of errors, we found differences between U-18 and U-21 (*z* = −2.307; *p* = 0.021), U-19 and U-20 (*z* = −2.024; *p* = 0.043), U-19 and U-21 (*z* = −2.095; *p* = 0.036), U-20 and U-21 (*z* = −4.133; *p* = 0.001), and U-20 and U-22 (*z* = −2.680; *p* = 0.007).

In the case of block, no significant gendered differences in accuracy were found (*z* = −0.881; *p* = 0.378) but a significantly higher number of errors were found in female players (*z* = −3.315; *p* = 0.001). Significantly higher accuracy was also found between U-21 and U-18 (*z* = −4.143; *p* = 0.001), U-19 (*z* = −3.218; *p* = 0.001), U-20 (*z* = −4.226; *p* = 0.001), and U-22 (*z* = −4.362; *p* = 0.001).

## Discussion

The aim of this study was to analyze gendered tactical and technical behavior across different ages in young, elite beach volleyball players. The findings show gendered and age variability and this should be taken into account in future training tasks.

Results show a constant work time and match time in women without differences between age. In men these values are not constant. They do not show differences with women but there is a decrease in time as the age is older. Studies such as Pérez-Turpin et al. (2009) show durations in rally, set, and match similar to those obtained in the older categories. This data can be related to the game dimensions (Giatsis et al., 2003) assigned to an individual space of play per player of 32 m. These data suggest that beach volleyball is a sport based on explosive actions of anaerobic alactic type.

The serve is a positive performance indicator of winning teams (Zetou et al., 2006; Medeiros et al., 2014, 2017). The results showed significant gendered differences in the effectiveness of the serve. This difference between men and women is a consequence of serve power values (Laios, 2008). Therefore, there are differences in accuracy and error between older teams in relation to the other teams. Koch and Tilp (2009) found that the different types of serve (jump serve, float serve, and jump float serve) do not show effectiveness differences in terms of the opposing team’s reception in elite female players. It is worth noting that the float serve is the most common technique used by women players (Tilp et al., 2006). In this case, no found differences in effectiveness of the different types of serve in women. In men, effectiveness decreases when players serve in motion and using more power. Thus, male players favor a float jump serve given that it causes difficulty for opponents through ball oscillation (Buscà et al., 2012; Medeiros et al., 2014). The results show that the jump serve is the one with the highest percentage of errors and produces the fewest aces, thus being in line with Laios (2008) in terms of errors but not in terms of aces. Koch and Tilp (2009) showed that the jump serve produced the most aces but with high levels of error. In the same line, Michalopoulou et al. (2005) found that the winning teams in the Greek league made fewer serve errors and prevented opponents from attacking in optimum conditions. Previous studies showed similar results (Palao and Ortega, 2015; Šimac et al., 2017). These data are in line with our results as they find differences according to players’ age. Experience seems to be an important factor for managing tough environments (fatigue, stress, losing, etc.).

Setting is one of the techniques that characterize successful players (Šimac et al., 2017). Results show differences between the use of forearm pass and overhand pass in women and men. Women use forearm pass more times than men. Results between age groups show a trend toward a greater use of forearm pass in younger age and overhand pass categories in the older ones. The available data show that the most frequent type of setting is a forearm pass. Similar tendencies have been found in other studies (Tilp et al., 2006; Medeiros et al., 2014). Tilp et al. (2006) and Koch and Tilp (2009) show that the overhand pass is more used by senior male players while the forearm pass is more frequent in senior women. The difference in the type of setting used is not the same as effectiveness, the overhand pass being more effective both in young players (González-Silva et al., 2019) and in male and female players (Tilp et al., 2006). This could be due to rule limitations and the environmental conditions inherent to beach volleyball.

Winning teams show higher efficiency in attack than losing teams (Michalopoulou et al., 2005; Afonso et al., 2012). Results show no differences between men and women, unlike Chinchilla-Mira et al. (2012) who found differences in effectiveness and attack area between men and women. Efficiency in attack varies across different ages, finding differences in effectiveness between the younger and older categories. Medeiros et al. (2017), compared U-19, U-21, and Senior players, and showed that losing teams made errors in attack (except for U-21). Furthermore, errors in attack define a higher difference in number of sets won (Giatsis and Zahariadis, 2008). Michalopoulou et al. (2005) found that the teams in the Greek league winning more points were those which made the fewer mistakes, were more effective in attack, and stopped opponents’ counterattacks, with no differences in attack organization. Giatsis and Tzetzis (2003) found that the difference between winning and losing teams was a high number of errors, though they depended on the type of attack used. Several factors can cause these differences, such as years of experience and styles of play (Papadimitriou et al., 2004; Gabbett and Georgie, 2007; Zapartidis et al., 2009; Sheppard et al., 2013). Furthermore, high-level players have the ability to execute different types of actions and display adaptable performance and self-organized behavior (Davids et al., 2013).

Good blocking is also a performance indicator in terms of winning or losing; winning teams achieved a higher number of points in this game action (Grgantov et al., 2005; Medeiros et al., 2014, 2017). Results show a greater number of errors in women’s blocks. Koch and Tilp (2009) classified blocks into fake blocks, which seem to be more appropriate in women’s competition, and blocks, more used by men and characterized by energetic activity at the net. In the same line, Laios (2008) also found that women significantly favor the use of fake blocks. Tilp et al. (2006) found similar results in terms of fake blocks due to a higher use of shots on the line, which are easier to defend in the court. Because of this, women may not be familiar with the use of blocks. Women show a higher number of errors when compared to men, as opposed to the results obtained by Laios (2008), who found no effective differences in blocks. Regarding age groups, the U-21 group presents differences in the effectiveness of blocks with the other groups. These results may be due to the experience of the players in game. The differences in terms of blocks might be related to a series of factors such as limited technical ability (Smith et al., 1992; Lobietti and Merni, 2006), bad reception (Michalopoulou et al., 2005; Koch and Tilp, 2009), jump height (Sheppard et al., 2009; Sterkowicz-Przybycien et al., 2014), and strategy (Schläppi-Lienhard and Hossner, 2015).

## Conclusion

This paper provides reference values on temporal, technical, and tactical variables and efficacy in the different categories that can be used in training design. Temporal variables allow a better understanding of the duration of efforts in the different categories. Block, set, and serve variables show differences in gender and age. Although it is necessary to have a specific skill level in order to participate in the best international tournaments, some aspects can be studied and learned by players. This results can provide a chance of developing training programs to help the young national teams increasing the competitive level of international beach volleyball. We thus need more studies about players’ development in order to understand beach volleyball.

## Data Availability Statement

The datasets generated for this study are available on request to the corresponding author.

## Author Contributions

All authors listed have made a substantial, direct and intellectual contribution to the work, and approved it for publication.

## Conflict of Interest

The authors declare that the research was conducted in the absence of any commercial or financial relationships that could be construed as a potential conflict of interest.
