# Corrigendum: Performance Indicators in Young Elite Beach Volleyball Players

**DOI:** 10.3389/fpsyg.2020.00237

**Published:** 2020-02-19

**Authors:** José Antonio Pérez-Turpin, Luis María Campos-Gutiérrez, Carlos Elvira-Aranda, María José Gomis-Gomis, Concepción Suárez-Llorca, Eliseo Andreu-Cabrera

**Affiliations:** Research Group on Physical Activity Sciences and Sport, University of Alicante, Alicante, Spain

**Keywords:** beach volleyball, technical, match analysis, gender, age group

In the original article, the reference for “George and Panagiotis, 2008 is incorrect”. It should be “Giatsis and Zahariadis, 2008”. Citations of George and Panagiotis, 2008 have been replaced and it has been removed from the reference list. “Giatsis and Zahariadis, 2008” is already in the reference list.

The authors apologize for this error and state that this does not change the scientific conclusions of the article in any way. The original article has been updated.

